# Four new species of *Oidardis* Hermann, 1912 (Diptera, Asilidae, Laphriinae, Atomosiini) from two major faunistic surveys in the Atlantic Rainforest

**DOI:** 10.3897/zookeys.350.6096

**Published:** 2013-11-14

**Authors:** Lucas A. Cezar, Eric M. Fisher, Carlos J. E. Lamas

**Affiliations:** 1 Museu de Zoologia, Universidade de São Paulo. Av. Nazaré, 481, São Paulo, SP, 04263-000 Brazil; 2 Programa de Pós-Graduação em Entomologia, Faculdade de Filosofia, Ciências e Letras de Ribeirão Preto, Universidade de São Paulo, Brazil; 3 El Dorado Hills, California; Research Associate, California State Collection of Arthropods, Sacramento, California

**Keywords:** Robber-flies, taxonomy, Brazil, Neotropical

## Abstract

Two recent faunistic surveys in the Brazilian Atlantic Forests region, the PROFAUPAR and the Biota/FAPESP Program, have provided important material for the discovery of new taxa from Brazil. We describe herein four new species of robber-flies of the genus *Oidardis* (*O. falcimystax* **sp. n.**, *O. fontenellei* **sp. n.**, *O. maculiseta* **sp. n.** and *O. marinonii* **sp. n.**), including illustrations and details on male hypopygia and female genitalia. A distribution map and a key to the species of *Oidardis* from the Brazilian Atlantic Forests region, including *O. triangularis* (Hermann), 1912, are also provided.

## Introduction

The Atomosiini are small compact assassin flies, whose length can range from 4–12 mm. Although they seem to be the dominant group of Asilidae in Neotropical forested areas, they are easily overlooked in the field due to their small size and usual preference for shadowy environments (Fisher 1985, 2009).

*Oidardis* Hermann is characterized by a smoothly curved lateral eye margin, scutum densely covered by short erect setulae and the absence of spines on the scutellum (Artigas et al. 1991, Fisher 2009). Two groups of species are clearly noted, based on the degree of sexual dimorphism. Males of the highly dimorphic species bear a rather long modified seta on the hind tibiae, related to the elaborate courtship behaviour they display. Species of the less dimorphic group display a much plainer sexual behaviour (Fisher 1985, 2009, Fisher and Hespenheide 1992), and the males lack the long setae on the posterior tibiae.

*Oidardis* comprised seven species prior to this study, distributed from Costa Rica to Brazil. They are almost exclusively found perching on twig tips in shaded understory. Five of them occur in the dense forests in the Amazon (*Oidardis aenescens*, *Oidardis aveledoi*, *Oidardis curupaoensis* and *Oidardis gibbosa*) and Central America (*Oidardis gibba*). For the Atlantic Forest, and Brazil as a whole, there were only two species recorded in previous works, *Oidardis triangularis* (Hermann), 1912 and *Oidardis nigra* (Hull), 1962 (Hull 1962, Artigas et al. 1991, Fisher 2009).

The Atlantic Rainforest is one of the world’s highest diversity biomes, with a large number of endemic species; yet, it is also one of the most devastated biomes due to human occupation and development. Undisturbed habitat occupies less than 7% of the original area, and it is recognized as one of the world’s hotspots for conservation (Da Fonseca 1985, Morellato and Haddad 2000, Myers et al. 2000).

In this scenario of high diversity and intense destruction, providing more knowledge on this biome is a matter of great urgency. Two major efforts seeking a wider consciousness about the insect diversity of the Brazilian Atlantic Rainforest should be noted: the projects BIOTA/FAPESP and PROFAUPAR.

The BIOTA program, funded by the São Paulo State Research Foundation (FAPESP), was primarily aimed at surveying and characterizing São Paulo state biodiversity, and guiding actions for its conservation. Since the Atlantic Forest is the most representative biome in São Paulo, it has attracted most of the effort of this survey. Some projects within the program also included expeditions to Atlantic Forest areas outside São Paulo state. Over 500 researchers were included in more than 70 projects in BIOTA/FAPESP program (Staley 2001, Metzger and Casatti 2006).

Another remarkable effort at increasing knowledge of Brazilian biodiversity was the Survey of the Entomological Fauna of Paraná State, PROFAUPAR, initiated by Dr. Renato Contin Marinoni. It was conducted from 1986 to 1988 and focused on Paraná state biodiversity in the different ecosystems that occur in its area (Marinoni and Dutra 1991).

Presented here are the illustrated descriptions, with details on male terminalia and female genitalia, of four new species of *Oidardis* from the Atlantic Forest, collected under BIOTA/FAPESP and PROFAUPAR faunistic surveys. These descriptions represent three species of the highly dimorphic group in the genus (*Oidardis falcimystax* sp. n., *Oidardis fontenellei* sp. n., and *Oidardis maculiseta* sp. n.), and one of the less dimorphic group (*Oidardis marinonii* sp. n.). A distribution map and a key to identification of all species of *Oidardis* occurring in this biome are also provided. The following results are part of a wider ongoing research on this genus. Fauna occurring in other biomes will be included in future publications.

## Material and methods

Specimens were examined with a ZEISS Stemi SV6 Stereomicroscope. Terminalia of selected paratypes were dissected and cleared in KOH at 25°C for 24 hours; dehydrated under an alcoholic series, in increasing concentration (30–95%); examined in temporary slides with glycerine; drawn under microscope with aid of a ZEISS Axioskope 40 *camera lucida*; and stored in a plastic microtube pinned with the specimen. Descriptions of the holotypes include a discussion of intraspecific variation; descriptions were generated with the software package “CSIRO DELTA (Description Language for Taxonomy) for Windows” v. 1.04 (Dallwitz 1980, Dallwitz et al. 1999). Mantis v. 2.0.1 (Naskrecki 2008) was used as a database, primary generator for material examined lists, and exporting locality data to Google Earth v. 6.1.0.5001. Distribution maps were prepared in Quantum GIS v. 1.7.0 Wroclaw, from locality data imported from Google Earth; ecorregion shapefiles for the maps follow Olson et al. (2001) and were obtained from World Wildlife Fund (WWF) website (World Wildlife Fund 2012).

The identification key includes the four new species of *Oidardis*, plus *Oidardis triangularis*. *Oidardis nigra*, thought probably to be a junior synonym of *Oidardis triangularis*, remains *inquirenda* since the type-material is lost; *Oidardis nigra* is not included in the dichotomous key to species of *Oidardis*.

Photographs were taken under ZEISS Discovery V20 Stereomicroscope with a ZEISS AxioCam Mrc5 camera attached, connected to a desktop computer through ZEISS AxioVs40 v. 4.8.2.0 software. Image stacks were assembled in Combine ZP software (Hadley 2010). Adobe Photoshop was used for editing images. Line drawings and plates were prepared in the Adobe Illustrator software.

Terminology for general morphology follows Cumming and Wood (2009); Stuckenberg (1999) for antennal structures; and Theodor (1976) for characters of male terminalia not mentioned in Cumming and Wood (2009). Tergites are often referred to as “T” followed by the segment number (e.g. T3 for the tergite of the third abdominal segment).

Depository for the specimens is noted within parentheses under the material examined section, according to acronyms listed below:

BMNH British Museum (Natural History), London

CNC Canada National Collection of Insects, Arachnids and Nematodes, Ottawa

DZUP Coleção Entomológica Padre Jesus Santiago Moure, Departamento de Zoologia, Universidade Federal do Paraná, Curitiba

EFISHER Eric Fisher’s private collection, El Dorado Hills

LEEID Laboratório de Ecologia Evolutiva de Insetos de Dossel, Universidade Federal de Ouro Preto, Ouro Preto

MNRJ Museu Nacional, Universidade Federal do Rio de Janeiro, Rio de Janeiro

MZUEFS Museu de Zoologia, Universidade Estadual de Feira de Santana, Feira de Santana

MZUSP Museu de Zoologia, Universidade de São Paulo, Sao Paulo

ZSM Zoologische Staatssammlung München, Munich

## Results

### Identification key to the Atlantic Forest species of *Oidardis* Hermann (except for *Oidardis nigra*, which is unrecognized)

**Table:** 

1	Gibbosity extending through lower half of face or beyond (Fig. 5A); abdomen cup-shaped; legs yellow or light-brown. Males with modified mystax (pair of regular setae dorsally, pair of dark-brown laterally-flattened setae medially, and pair of white, sinuous, filiform setae ventrally) (Fig. 5A); modified tibial setae dark-brown, shorter than femur, with dark-brown leaf-shaped, longitudinally-striated lamella on apical 1/5, inserted on middle of hind tibiae (Fig. 2A) [Peru, Bolivia, Brazil (Goiás, Mato Grosso, São Paulo and Paraná) and Argentina]	*Oidardis falcimystax* sp. n.
–	Gibbosity extending through lower third of face at most (Figs 5B–D). Males with only regular mystacal setae (Figs 5B–D). Other combination of characters	2
2(1)	Body yellow and black or light-brown and black (Figs 1C–D, G–H); anterior region of scutum, pleura and lateral margins of tergites yellow	3
–	Body entirely black or dark-brown (Figs 1A–B, E–F); anterior region of scutum, pleura and lateral margins of tergites dark-brown	4
3(2)	Scutum vestiture homogeneously directed (all setulae reclinate, including posterior region of scutum); antenna usually entirely dark-brown; anterior and mid femora brown. Male with hind femur yellow; hind tibia entirely dark-brown; with modified tibial seta on posterior leg (short light-brown seta, with slightly dilated apex white) (Fig. 2B) [Brazil (Sergipe, Bahia, Minas Gerais, Espírito Santo, Rio de Janeiro, São Paulo, and Paraná)]	*Oidardis fontenellei* sp. n.
–	Scutum vestiture heterogeneously directed (setulae on posterior region of scutum proclinate); antenna with yellow or light-brown scape and pedicel; anterior and mid femora yellow. Male with hind femur dark-brown; hind tibia yellow dorsally and dark-brown ventrally; without modified tibial seta on hind leg (Fig. 2D) [Brazil (São Paulo and Paraná)]	*Oidardis marinonii* sp. n.
4(2)	Legs predominantly yellow, femora dorsally and tibiae ventrally dark-brown (Fig. 2C); face with golden pollinosity (Fig. 4C). Male with modified tibial seta dark-brown, as long as femur, golf-club-shaped, with apical 1/4 as a large white lamella with black spot at apex (Fig. 2C); mystax short (Fig. 5C); mid prong of the phallus much longer than lateral prongs (Fig. 7C). [Brazil (Goiás, São Paulo and Paraná)]	*Oidardis maculiseta* sp. n.
–	Legs predominantly dark-brown, anterior and mid tibiae at most slightly lighter dorsally; face usually copper-pollinose, with gibbosity silver-pollinose (rarely entirely golden-pollinose). Male without modified tibial seta; mystax as long as, or longer than, proboscis; phallus with equal-sized prongs; gonocoxite with three spines in characteristic fork-like pattern on apex [Brazil (Minas Gerais, Espírito Santo, Rio de Janeiro, São Paulo, Paraná, and Santa Catarina)]	*Oidardis triangularis* (Hermann)

### Descriptions

#### Oidardis falcimystax sp. n.

http://zoobank.org/7659EB6E-08DA-4831-91BF-73C934C6E774

http://species-id.net/wiki/Oidardis_falcimystax

Figures 1A–B , 2A , 3A , 4A , 5A , 6A–C , 7A , 8C , 10

##### Diagnosis.

Gibbosity extending through lower half of face or beyond; abdomen cup-shaped; legs yellow or light-brown. Males with modified mystax (pair of regular setae dorsally, pair of dark-brown laterally-flattened setae and pair of white sinuous filiform setae ventrally); modified tibial setae dark-brown, shorter than femur, with dark-brown leaf-shaped, longitudinally-striated lamella on apical 1/5, inserted on middle of hind tibiae.

##### Description.

**Holotype.** Male. Body shiny black. Total length, excluding antennae, 5 mm; length of thorax, 1.2 mm; length of wing, 4.4 mm; greatest width of abdomen, 1 mm.

**Head, laterally.** Face, between antennal insertion and gibbosity, plane with eye margin; gibbosity prominent, equals ventral 0.7 of face height; dorsal occipital setae dark-brown, lateral occipital setae white, ventral occipital setae white; proboscis 0.53 × the height of head, with a pair of yellow macrosetae ventrally; palpus dark-brown, with yellow setae apically. **Antenna.** Antenna 0.74 × as long as the height of eye, entirely dark-brown, with dark-brown setae and macrosetae; antennal insertion at dorsal 0.2 of head height; scape slightly longer than pedicel, with medium-sized ventral seta, numerous short setae ventrally and around the whole segment apically; pedicel oval; postpedicel oblong, 1.7 × length of basal two segments, brown-pollinose, except for silvery-yellow pollinosity on elliptical sensorial area on inner face, with dorsal spine subapical (3/4 length of postpedicel or beyond). **Head, anteriorly.** Head 1.39 × as wide as high; face 0.14 × as wide as head, silvery-pollinose, except on shiny upper half of gibbosity; mystax long (extending beyond the apex of proboscis), comprised of 6 macrosetae – dorsal pair regular and dark-brown, middle pair spatulate and dark-brown, and ventral pair sinuous-filiform and white; facial setae, other than mystax, pale-yellow; frons golden-pollinose; orbital setae dark-brown; vertex golden-pollinose; ocellar tubercle golden-pollinose, as high as vertex, 0.29 × as wide as frons, anterior ocellus 0.11 × as wide as frons by the ocellus position.

**Thorax.** Postpronotal lobe dark-brown; scutum shiny black, not punctate, vestiture golden, equal-sized, longest setae as long as half the scape, reclinate anteriorly and proclinate posteriorly; one dark-brown notopleural; scutellum black, scutellar margin strongly impressed, marginal scutellar macrosetae dark-brown, equal-sized, longest ones much shorter than scutellum (as long as the width of the rim); postalar callosity dark-brown, partly with bright-blue reflections; pleuron shiny dark-brown, with silvery-white pollinosity; setulae on proepisternum, katepisternum and anepisternum yellow; one anepisternal macroseta, plus fine setulae, yellow; tuft of katatergal macrosetae light-brown; anatergite with golden, hair-like setae.

**Legs.** Coxae orange-yellow; trochanter orange-yellow, with fine yellow setulae; femora yellow, slightly darkened dorsally—except hind femur, only darkened distally, covered with short stout yellow setulae dorsally, with dark setae on apical 1/3 dorsally, hind femur with 4 long yellow ventral macrosetae in a row along proximal half; tibiae entirely yellow, with yellow setulae, long yellow macrosetae and thick spines; hind tibia entirely covered by golden setulae, with white setulae ventrally, long dark-brown macrosetae ventrally and long dark-brown macrosetae anterodorsally; modified tibial setae attached to hind tibia at middle, dark-brown, shorter than femur, with dark-brown leaf-shaped longitudinally-striated lamella on apical 1/5; tarsi yellow, 5th tarsomere dark-brown, with stout yellow setae dorsally, and densely covered with thick spine-like golden setae, 5th tarsomere with 3 setae apically, opposite the claws and longer than them; claws yellow on base and black apically; pulvilli yellow and fringed; empodium shorter than claws.

**Wing.** Brownish, darker along upper margin; cell r_1_ with short slightly-concave stalk (2 × the length of r-m); crossvein r-m at proximal half of cell d, aligned to the end of Sc; cell m_3_ narrowing distally (M_2_ and M_3_ converging by the end of cell m_3_), with stalk slightly longer than r-m, apex of m_3_ and apex of cell d parallel and unaligned, apex of m_3_ beyond apex of d; crossvein bm-cu long, base of M_3_ and CuA_1_ distant from each other and not appearing as an “X”; cell cu*p* with stalk shorter than r-m; posterior margin of wing slightly convex at distal half; calypters orange, with light-brown margin and fringe of short brown setae; halter with orange stem, white knob.

**Abdomen.** Black, punctate, with sides diverging posteriorly, T2 1.9 × wider than long; vestiture longer and lighter laterally and ventrally, several white macrosetae present on lateral margin of T1 and T2. **Male terminalia.** Hypopygium barely conspicuous; hypandrium regular-sized (2/3 the width of hypopygium or more), much wider than long, anterior margin straight to slightly convex, posterior margin sharply pointed; gonocoxites free, gonocoxal prolongation thin, smoothly curved inwards, with 2 spines at apex; gonostylus reduced, round, laterally flattened, free, attached to the base of gonocoxite; apex of phallus with three equal-sized prongs; epandrium straight in lateral view; lobes of hypoproct short.

**Female.** Total length, excluding antennae, 5.3–6.2 mm, (n=5); length of thorax, 1.4–1.7 mm, (n=5); length of wing, 4.6–5.4 mm, (n=5); greatest width of abdomen, 1.0–1.3 mm, (n=3). Differs from male as follows: gibbosity that equals ventral 0.5–0.64 of face height; proboscis 0.48–0.55 × the height of head; antenna 0.68–0.8 × as long as the height of eye; antennal insertion at dorsal 0.22–0.4 of head height; postpedicel 1.4–1.5 × length of basal two segments; head 1.3–1.43 × as wide as high; face 0.11–0.14 × as wide as head; mystax comprised of regular golden-brown macrosetae; ocellar tubercle 0.28–0.37 × as wide as frons; anterior ocellus 0.13–0.17 × as wide as frons by the ocellus position; hind tibiae with fine, medium-sized, golden setae ventrally, long, yellow macroseta inserted ventrally on the middle, and long, dark-brown macrosetae anterodorsally; modified tibial setae absent; T2 2.13–2.29 × wider than long; **Female genitalia.** Three spermathecae; reservoirs cylindrical, disposed in a spiral; spermathecal ducts opening independently at the bursa; genital fork rectangular, U-shaped, arms anteriorly thick, posteriorly truncate, divergent; accessory glands undistinguishable.

**Morphological variation.** Total length, excluding antennae, 5.0–6.3 mm, (n=10); length of thorax, 1.2–1.5 mm, (n=10); length of wing, 4.2–4.9 mm, (n=9); greatest width of abdomen, 0.9–1.2 mm, (n=9). Some specimens differed from the holotype, as follows: gibbosity that equals ventral 0.5–0.7 of face height; lateral occipital setae dark-brown; proboscis 0.35–0.53 × the height of head; proboscis with dark-brown macrosetae, ventrally; antenna 0.7–0.8 × as long as the height of eye; antennal insertion at dorsal 0.2–0.27 of head height; numerous short setae on a row around the scape; postpedicel 1.4–1.8 × length of basal two segments; postpedicel golden-pollinose; head 1.22–1.43 × as wide as high; face 0.07–0.21 × as wide as head; frons silvery-pollinose; orbital setae golden-brown; ocellar tubercle 0.29–0.33 × as wide as frons; anterior ocellus 0.11–0.14 × as wide as frons by the ocellus position; postpronotal lobe black with yellow spot dorsal to mesothoracic spiracle; scutal vestiture dark-brown; longest marginal scutellar macrosetae shorter than scutellum; postalar callosity light-brown; fore and mid tibiae with yellow setulae, long dark-brown macrosetae, and thick spines; tarsi with stout dark-brown setae dorsally and densely covered with thick spine-like golden setae; claws reddish on base and black apically; calypters white, with light-brown margin and fringe of short yellow setae; halter knob pale-yellow; T2 1.63–2.13 × wider than long.

##### Distribution.

Peru, Bolivia, Brazil (Goiás, Mato Grosso, São Paulo and Paraná) and Argentina.

##### Remarks.

This species share with an undescribed species from Panama–”*Oidardis signaseta*” Fisher (*nomen nudum*) (Fisher 2009: pp. 600, 604, 624, figs 46, 77)–several peculiar characters regarding the shape of gibbosity and mystax. The main difference between those two species is that “*Oidardis signaseta*” males bear two pairs of modified blade-shaped setae, whilst *Oidardis falcimystax* males bear only one pair, along with a pair of white sinuous filiform setae.

*Oidardis falcimystax*, in its farther western occurrences, inhabit Peruvian and Bolivian Amazon forests, as do its congeners *Oidardis aenescens* Hermann, 1912, and *Oidardis gibbosa* Hermann, 1912; yet, due to the singular morphology of its facial gibbosity and mystax, and the modified tibial seta, *Oidardis falcimystax* can be readily distinguished from these other two species. *Oidardis aenescens* have similar size and overall coloration, and even presents a similarly-prominent gibbosity, but its extent reaches no more than the ventral third of the face; besides, males of this species do not present any striking modification on mystax or tibial setae. *Oidardis gibbosa* presents a completely different color pattern, along with distinctly modified tibial seta, among other characters. *Oidardis aenescens* and *Oidardis gibbosa* will be thoroughly presented in future publications.

This species, along with *Oidardis maculiseta*, is the first occurrence of *Oidardis* in the Cerrado area, since the genus is almost exclusively found in dense-forest biomes. Those occurrences are, though, probably related to the higher forest environments in Cerrado, “Cerradão”, and riparian forests, which have been noticed to share fauna of the Lower Diptera, at least, with Atlantic Semi-deciduous Forest areas (D. S. Amorim, unpublished data).

##### Etymology.

From the Latin, *falx* = scythe, and the Greek, *mystax* = moustache. Refers to the flattened blade-shaped mystacal macrosetae.

##### Type-material examined.

**Holotype: Brazil:** São Paulo, Sertãozinho, elev. 550 m (21°9.14'S, 48°5.72'W), 10–24.xi.2010, coll. V. C. Silva & P. F. Donda (Frag. 2) - male (MZUSP) **Paratypes: Argentina:** Misiones, Puerto Iguazu, behind Hotel Orquídeas, (25°37'29.59"S, 54°33'2.65"W), 1–6.ii.1992, coll. S.A. Marshall - 1 female (EFISHER); **Bolivia:** Beni, Palos Blancos, elev. 600 m (15°35'S, 67°15'0"W), 11–15.i.1976, coll. L.E. Pena - 2 males (CNC); **Brazil:** Goiás, Jataí, (17°52'33.25"S, 51°43'17.19"W), xi.1972, coll. F. M. Oliveira - 1 female (MZUSP); Mato Grosso, Chapada dos Guimarães, (15°27'10.26"S, 55°44'21.02"W), 21.xi.1983, coll. Exc. Dep. ZOO - 4 males (DZUP, MZUSP); same locality, 24.xi.1983, coll. Exc. Dep. ZOO - 1 male (DZUP); Paraná, Fênix, Reserva Estadual ITCF, (23°55'0.05"S, 51°57'38.26"W), 24.xi.1986, coll. Lev. Ent. PROFAUPAR - 1 male (MZUSP); same locality, 8.xii.1986, coll. Lev. Ent. PROFAUPAR - 5 males (DZUP); same locality, 15.xii.1986, coll. Lev. Ent. PROFAUPAR - 2 males (DZUP); same locality, 22.xii.1986, coll. Lev. Ent. PROFAUPAR - 5 males (DZUP); same locality, 29.xii.1986, coll. Lev. Ent. PROFAUPAR - 5 males (DZUP); Foz do Iguaçu, (25°32'48.83"S, 54°35'17.42"W), 3.xii.1966, coll. Exc. Dep. ZOO - 1 female (DZUP); same locality, 5.xii.1966, coll. Exc. Dep. ZOO - 1 female (DZUP); same locality, 7.xii.1966, coll. Exc. Dep. ZOO - 1 female, 3 males (incl. 4 paratypes) (DZUP, MZUSP); same locality, 12.xii.1966, coll. Exc. Dep. ZOO - 3 males (DZUP); São Paulo, Sertãozinho, elev. 550 m (21°9.14'S, 48°5.72'W), 19.i.2011, coll. V. C. Silva & P. F. Donda (Frag. 1) - 1 male (MZUSP); same locality, 2.ii.2011, coll. V. C. Silva & P. F. Donda (Frag. 1) - 1 female (MZUSP); same locality, 16.ii.2011, coll. V. C. Silva, P. F. Donda, G. P. Ignácio & D. S. Amorim (Frag. 1) - 1 female (MZUSP); **Peru:** Madre de Diós, Tambopata Reserve - 30 km SW Puerto Maldonado, (12°44'50.9"S, 69°25'46.46"W), 6.xii.1982, coll. J.J. Anderson - 1 male (EFISHER).

**Figure 1.**
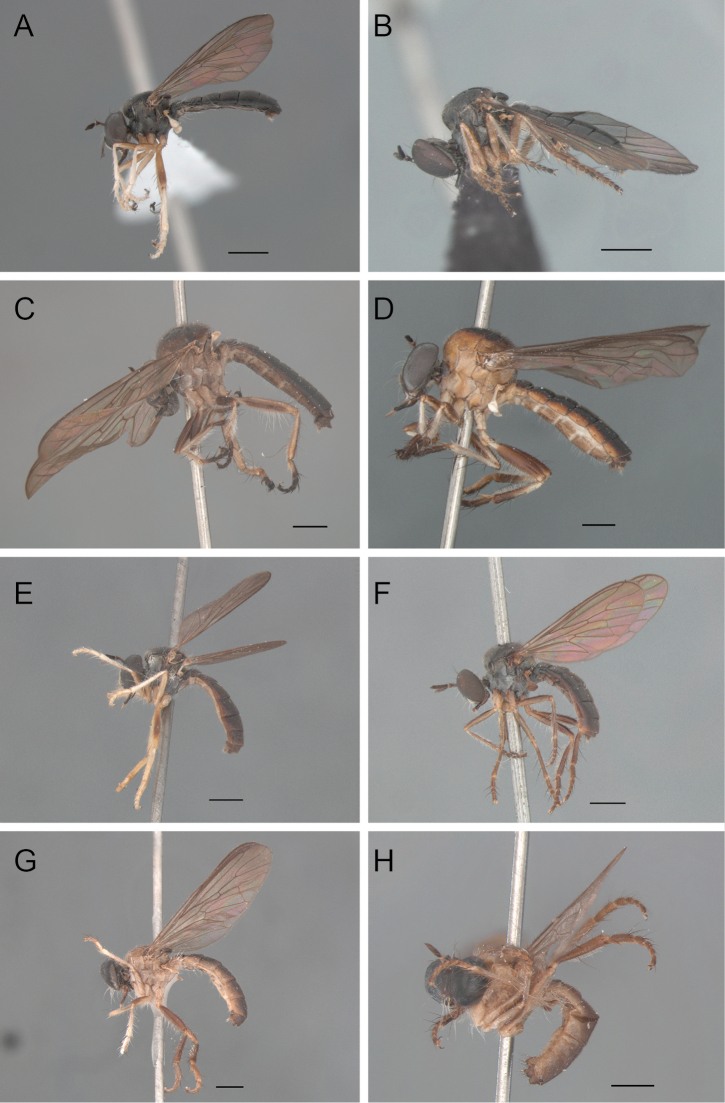
Habitus, lateral view: **A** *Oidardis falcimystax* male (HT) **B** female (PT) **C** *Oidardis fontenellei* male (HT) **D** female (PT) **E** *Oidardis maculiseta* male (HT) **F** female (PT) **G** *Oidardis marinonii* male (HT) **H** female (PT). Scale bar = 1 mm.

**Figure 2.**
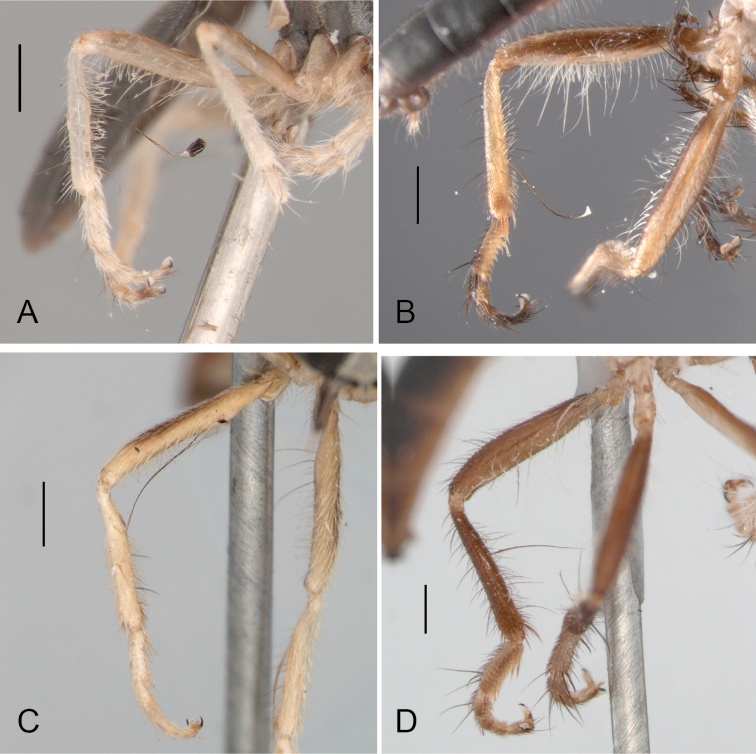
Hind leg, male: **A** *Oidardis falcimystax* (PT) **B** *Oidardis fontenellei* (HT) **C** *Oidardis maculiseta* male (HT) **D** *Oidardis marinonii* (HT). Scale bar = 0.5 mm.

**Figure 3.**
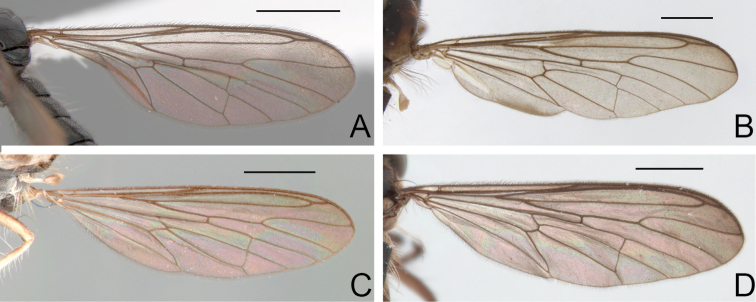
Wing, male: **A** *Oidardis falcimystax* (HT) **B** *Oidardis fontenellei* (PT) **C** *Oidardis maculiseta* (HT) **D** *Oidardis marinonii* (PT). Scale bar = 1mm.

**Figure 4.**
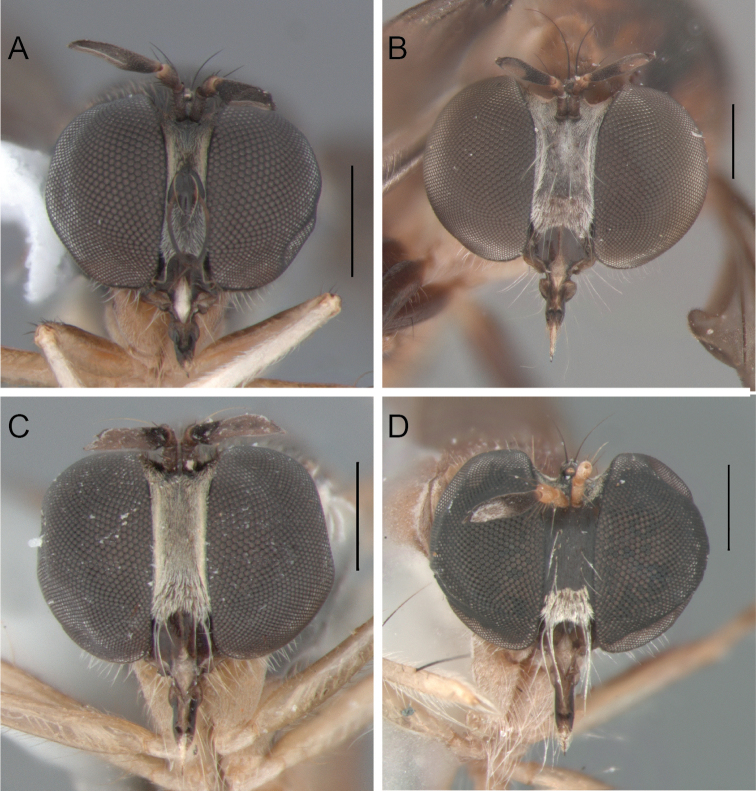
Head, male, anterior view: **A** *Oidardis falcimystax* (HT) **B** *Oidardis fontenellei* (HT) **C** *Oidardis maculiseta* male (HT) **D** *Oidardis marinonii* (HT). Scale bar = 0.5mm

**Figure 5.**
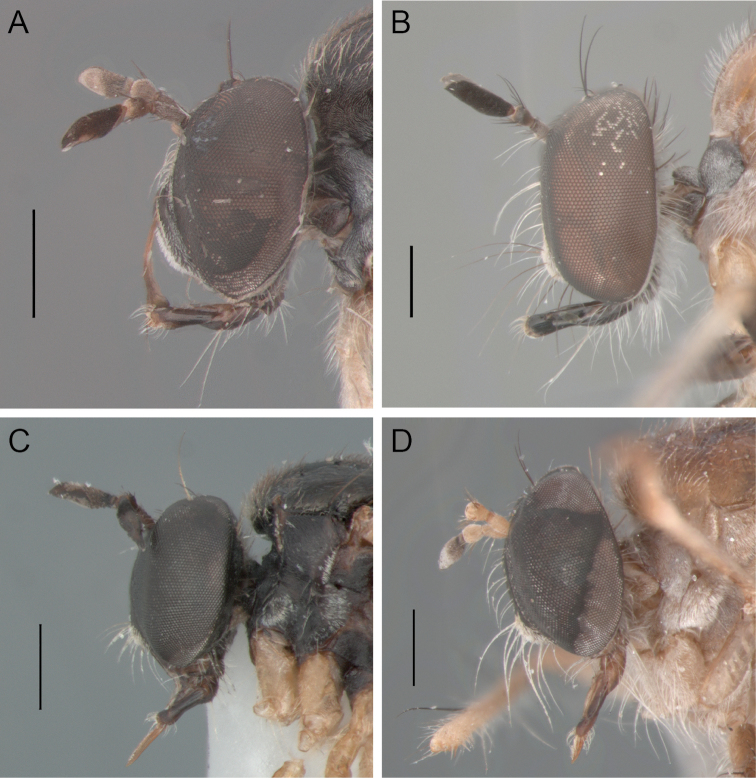
Head, male, lateral view: **A** *Oidardis falcimystax* (HT) **B** *Oidardis fontenellei* (PT) **C** *Oidardis maculiseta* (PT) **D** *Oidardis marinonii* (HT). Scale bar = 0.5 mm.

**Figure 6.**
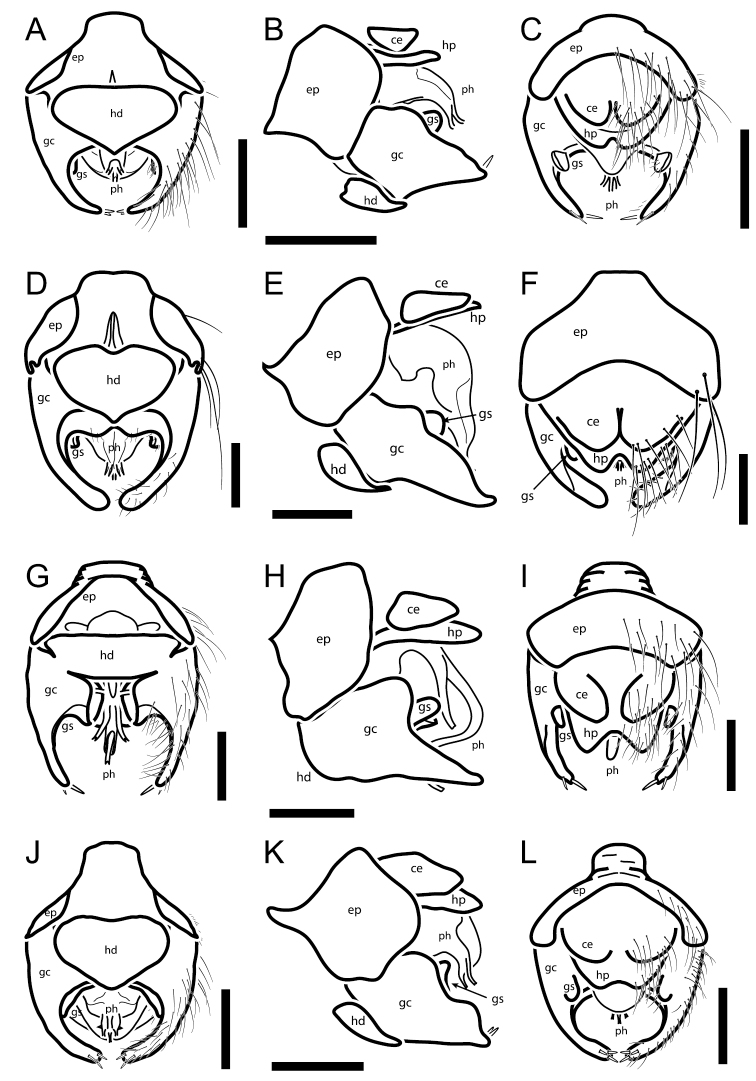
Male terminalia: **A–C** *Oidardis falcimystax* **A** ventral **B** lateral and **C** dorsal views **D–F** *Oidardis fontenellei* **D** ventral **E** lateral and **F** dorsal views **G–I** *Oidardis maculiseta* **G** ventral **H** lateral and **I** dorsal views **J–L** *Oidardis marinonii* **J** ventral **K** lateral and **L** dorsal views. *ep*, epandrium; *gc*, gonocoxite; *gs*, gonostylus; *hp*, hypandrium; *ph*, *phallus*. Scale bar: 0.2 mm.

**Figure 7.**
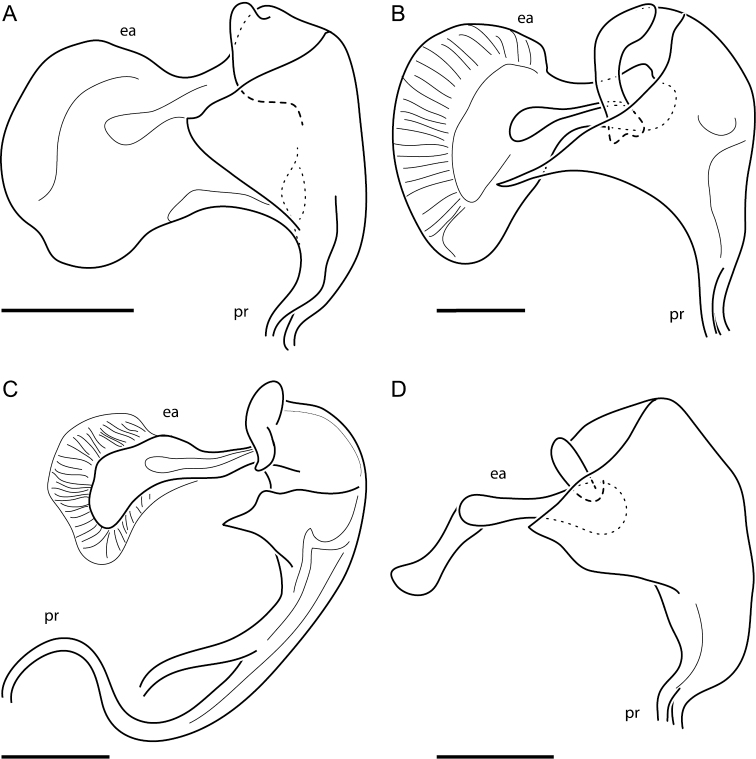
*Phalli*, lateral view: **A** *Oidardis falcimystax* **B** *Oidardis fontenellei* **C** *Oidardis maculiseta* male **D** *Oidardis marinonii*; *ea*, ejaculatory apodeme; *pr*, prongs. Scale bar: 0.1 mm.

**Figure 8.**
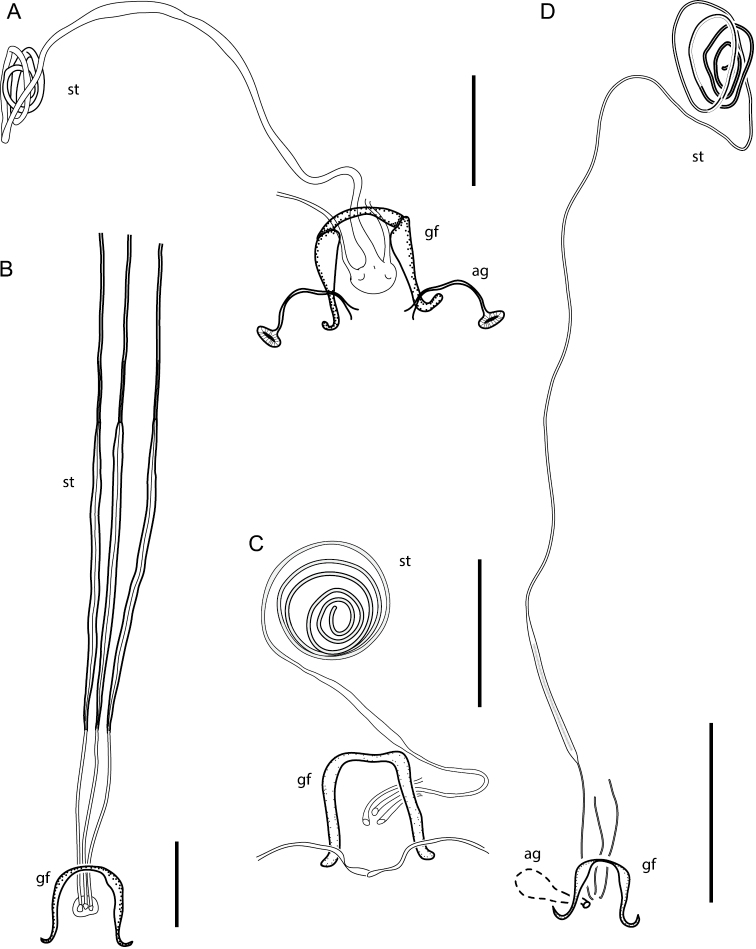
Spermathecae: **A** *Oidardis maculiseta* **B** *Oidardis fontenellei* **C** *Oidardis falcimystax* **D** *Oidardis marinonii*. *ag*, acessory gland; *gf*, genital fork; *st*, spermatheca. Scale bar: 0.2 mm.

#### Oidardis fontenellei sp. n.

http://zoobank.org/19052A06-27EA-4A04-B809-8F3973AB2625

http://species-id.net/wiki/Oidardis_fontenellei

Figures 1C–D , 2B , 3B , 4B , 5B , 6D–F , 7B , 8B , 9 , 10

##### Diagnosis.

Body shiny black and yellow; scutum with distinct arrow-like color pattern, in dorsal view; tergites with slightly paler to yellow lateral margins. Male with characteristic modified tibial seta, short light-brown with very slightly-dilated white apex.

##### Description.

**Holotype.** Male. Body yellow and black. Total length, excluding antennae, 7.9 mm; length of thorax, 1.8 mm; length of wing, 6.9 mm; greatest width of abdomen, 1.3 mm.

**Head, laterally.** Face, between antennal insertion and gibbosity, slightly concave; gibbosity prominent; dorsal occipital setae dark-brown, lateral occipital setae dark-brown, ventral occipital setae white; proboscis 0.42 × the height of head, with a pair of yellow macrosetae ventrally; palpus dark-brown, with yellow setae apically. **Antenna.** Antenna 0.76 × as long as the height of eye, entirely dark-brown, with dark-brown setae and macrosetae; antennal insertion at dorsal 0.18 of head height; scape slightly longer than pedicel, with long ventral seta, numerous short setae on a row around the segment; pedicel round; postpedicel lanceolate, 1.9 × length of basal two segments, golden-pollinose, except for coppery-yellow pollinosity on elliptical sensorial area on inner face, with dorsal spine subapical (3/4 length of postpedicel or beyond). **Head, anteriorly.** Head 1.6 × as wide as high; face 0.19 × as wide as head, silvery-pollinose; mystax long (extending beyond the apex of proboscis), comprised of 8 golden-brown macrosetae, and few shorter setae between the rows; facial setae, other than mystax, pale-yellow; frons silvery-pollinose; orbital setae dark-brown; vertex coppery-pollinose; ocellar tubercle silver-pollinose, as high as vertex, 0.21 × as wide as frons, anterior ocellus 0.09 × as wide as frons by the ocellus position.

**Thorax.** Postpronotal lobe yellow; scutum shiny dark-brown medially and yellow anteriorly and laterally in an arrow-like pattern, not punctate, vestiture dark-brown, equal-sized, longest setae as long as scape, uniformly reclinate; one dark-brown notopleural; scutellum black, scutellar margin smoothly marked, marginal scutellar macrosetae dark-brown, unequal-sized, longest ones slightly longer than scutellum; postalar callosity yellow, partly with bright-blue reflections; pleuron yellow, with silvery-white pollinosity; setulae on proepisternum, katepisternum, and anepisternum golden; two anepisternal macrosetae, plus fine setulae, dark-brown; tuft of katatergal macrosetae dark-brown; anatergite with dark-brown, hair-like setae.

**Legs.** Coxae orange-yellow; trochanter yellow, with fine yellow setulae; femora reddish-brown – except hind femur yellow, darkened anteriorly and posteriorly—covered with short stout brown setulae dorsally, with dark setae on apical 1/3 dorsally, ventrally with weak yellow setae in two rows, hind femur with 4 long yellow ventral macrosetae in a row along distal half; anterior four tibiae entirely yellow, with white setulae, long dark-brown macrosetae and thick spines; hind tibiae orange, entirely covered by dark-brown setulae, with stout dark-brown setulae, long dark-brown macrosetae anterodorsally; modified tibial setae attached to hind tibia at apical 1/3, light-brown, elongated and curved, tape-like flattened, with apical 1/7 translucent and slightly dilated; tarsi dark-brown, with stout dark-brown setae dorsally and densely covered with thick spine-like dark-brown setae, hind tarsi with flattened claw-like dark-brown setae dorsally, 5th tarsomere with 3 setae apically, opposite the claws and longer than them; claws dark-brown on base and black apically; pulvilli yellow and fringed; empodium shorter than claws.

**Wing.** Brownish, darker along upper margin and posteriorly on anal lobe; cell r_1_ with long slightly-concave stalk (4 × the length of r-m); crossvein r-m medially in cell d, aligned to the end of Sc; cell m_3_ parallel-sided distally (M_2_ and M_3_ parallel by the end of cell m_3_), with stalk slightly longer than r-m, apex of m_3_ and apex of cell d parallel and unaligned, apex of m_3_ beyond apex of d; crossvein bm-cu long, base of M_3_ and CuA_1_ distant from each other and not appearing as an “X”; cell cu*p* with stalk slightly longer than r-m; posterior margin of wing slightly convex at distal half; calypters yellow, with light-brown margin and fringe of short brown setae; halter with yellow stem, orange knob.

**Abdomen.** Black, punctate, with sides nearly parallel, T2 1.5 × wider than long; vestiture longer and lighter laterally and ventrally, several light-yellow macrosetae present on lateral margin of T1, T2, and T3, one lateral marginal macroseta present on T4 and T5. **Male terminalia.** Hypopygium very conspicuous; hypandrium regular-sized (2/3 the width of hypopygium or more), much wider than long, anterior margin straight to slightly convex, posterior margin sharply pointed; gonocoxites free, gonocoxal prolongation blunt, smoothly curved inwards, with no spines at apex; gonostylus reduced, round, laterally flattened, free, attached to the base of gonocoxite; apex of phallus with three equal-sized prongs; epandrium straight in lateral view; lobes of hypoproct short.

**Female.** Body yellow and black. Total length, excluding antennae, 6.1–8.8 mm, (n=4); length of thorax, 1.6–2.3 mm, (n=4); length of wing, 6.0–7.5 mm, (n=4); greatest width of abdomen, 1.7–1.9 mm, (n=3). Differs from male as follows: gibbosity that equals ventral 0.27–0.35 of face height; proboscis 0.5–0.52 × the height of head; antenna 0.63–0.83 × as long as the height of eye; antennal insertion at dorsal 0.2–0.38 of head height; postpedicel oblong; postpedicel 2–2.1 × length of basal two segments; head 1.55–1.63 × as wide as high; face 0.18–0.21 × as wide as head; anterior ocellus 0.06–0.08 × as wide as frons by the ocellus position; hind femur entirely reddish-brown; hind tibiae brown on distal third dorsally, with white setulae ventrally, fine, medium-sized, dark-brown setae ventrally, long, dark-brown macroseta inserted ventrally on the middle, and long, dark-brown macrosetae anterodorsally; modified tibial setae absent; calypters with yellow margin and fringe of short yellow setae; abdomen with sides diverging posteriorly; T2 1.73–2.18 × wider than long. **Female genitalia.** Three spermathecae; reservoirs absent; spermathecal ducts opening independently at the bursa; genital fork rectangular, U-shaped, arms anteriorly thick, posteriorly slender, divergent; accessory glands undistinguishable.

**Morphological variation.** Total length, excluding antennae, 6.4–8.2 mm, (n=10); length of thorax, 1.7–2.2 mm, (n=10); length of wing, 6.3–7.7 mm, (n=9); greatest width of abdomen, 1.2–1.4 mm, (n=10). Some specimens differed from the holotype, as follows: face, between antennal insertion and gibbosity, plane with eye margin; gibbosity equals ventral 0.31–0.46 of face height; ventral occipital setae yellow; proboscis 0.42–0.55 × the height of head; antenna 0.61–0.99 × as long as the height of eye; light-brown scape and pedicel, dark-brown postpedicel; antennal insertion at dorsal 0.18–0.26 of head height; postpedicel 1.4–2.1 × length of basal two segments; head 1.35–1.63 × as wide as high; face 0.12–0.21 × as wide as head; face pale-yellow-pollinose or silvery-pollinose or golden-pollinose; mystax comprised of 8–10 macrosetae; orbital setae golden-brown; anterior ocellus 0.06–0.09 × as wide as frons by the ocellus position; scutal vestiture white anteriorly and dark-brown posteriorly, unequal-sized; two notopleural macrosetae; scutellar margin strongly impressed; three anepisternal macrosetae; tuft of katatergal macrosetae light-brown; anatergite with golden, hair-like setae; coxae yellow; trochanter with fine, yellow setulae, and dark brown seta dorsally; tibiae yellow dorsally and brown ventrally; tarsi reddish-brown, with claw-like dark-brown setae ventrally, stout dark-brown setae dorsally, and densely covered with thick spine-like dark-brown setae; pulvilli brown; empodium well developed; cell r_1_ with long, slightly concave stalk (3.5–4 times length of r-m); calypters white; white knob; abdomen black with yellow lateral margins; T2 1.41–1.92 × wider than long; several white; one lateral marginal macroseta present on T4, T5, T6, and T7.

##### Distribution.

Brazil (Sergipe, Bahia, Espírito Santo, Minas Gerais, Rio de Janeiro, São Paulo and Paraná).

##### Remarks.

Although this species shares with *Oidardis marinonii* the lighter color of the body, with thorax and abdomen yellow laterally, they differ in a very conspicuous character, the modified seta ventrally on the hind tibia of males—even though *Oidardis fontenellei* bears the most discrete modified tibial seta observed in the genus. *Oidardis fontenellei* males display an elaborate courtship behavior: it approaches the female, perching on a lower position of the same twig its target is found; the male then hovers shortly behind the female, touching it quickly several times. (Guilherme Ide & Julia Almeida, pers. comm.)

*Oidardis fontenellei* occur preferentially in the Coastal Atlantic Forest, through its whole extension. The species was also found in Atlantic Semi-deciduous Forests in Minas Gerais and Bahia states, in localities no farther than 250 km from the coastline.

##### Etymology.

Honors Dr. Julio Fontenelle, for his efforts on a long-term survey on Diptera at Parque Estadual do Rio Doce, the largest fragment of preserved Atlantic Rainforest in Minas Gerais state.

##### Type-material examined.

**Holotype: Brazil:** Minas Gerais, Marliéria, Parque Estadual do Rio Doce, Trilha Tereza, elev. 253 m (19°42'10.69"S, 42°30'45.97"W), 25.x.2001, coll. J.C.R. Fontenelle - male (MZUSP) **Paratypes: Brazil:** Bahia, Camacan, RPPN Serra Bonita II Faz. Paris, elev. 190 m (15°25'12"S, 39°32'33"W), 3–7.ii.2009, coll. Nihei, Figueiredo, Almeida & Cezar - 1 male (MZUSP); Itapetinga, (15°15'23.24"S, 40°15'27.49"W), xi.1969, coll. F. M. Oliveira - 3 females, 9 males (MNRJ); Porto Seguro, Veracel, (16°27'3.98"S, 39°3'52.57"W), 2–5.xii.2002, coll. I. Castro - 1 female (MZUEFS); Espírito Santo, Santa Tereza, Estação Biológica Santa Lúcia, elev. 867 m (19°58'37.3"S, 40°32'22.5"W), 9–12.iv.2001, coll. C.O. Azevedo & eq. - 1 female (MZUSP); São Mateus, (18°43'0.16"S, 39°51'33.8"W), i.1971, coll. P. C. Elias - 1 female (MZUSP); Minas Gerais, Marliéria, Parque Estadual do Rio Doce, Trilha Tereza, elev. 253 m (19°42'10.69"S, 42°30'45.97"W), 25.x.2001, coll. J.C.R. Fontenelle - 7 males (LEEID, MZUSP); same locality, 1.xi.2001, coll. J.C.R. Fontenelle - 3 females, 1 male (LEEID, MZUSP); same locality, 8.xi.2001, coll. J.C.R. Fontenelle - 3 females, 3 males (LEEID, MZUSP); same locality, 9.xi.2003, coll. J.C.R. Fontenelle - 1 female (MZUSP); same locality, 31.x.-5.xi.2010, coll. J. C. Almeida & G. Ide - 6 females, 7 males (BMNH, MZUSP, ZSM); Paraná, Antonina, Reserva Sapitanduva, (25°25'46.77"S, 48°42'42.49"W), 1.xii.1986, coll. Lev. Ent. PROFAUPAR - 1 male (DZUP); same locality, 29.xii.1986, coll. Lev. Ent. PROFAUPAR - 1 male (DZUP); Rio de Janeiro, Nova Iguaçu, Reserva Biológica do Tinguá, (22°34'37"S, 43°26'6.6"W), 5–8.iii.2002, coll. S.T.P. Amarante & eq. - 2 males (MZUSP); Rio de Janeiro, (22°54'12.74"S, 43°12'34.51"W), i.1939, coll. Serviço Febre Amarela - 2 males (MZUSP); Rio de Janeiro, Jacarepaguá, Represa Rio Grande, (22°57'S, 43°21'0"W), xii.1969, coll. M. Alvarenga - 6 males (MNRJ); São Paulo, Bertioga, Praia de Guaratuba, (23°45'49.44"S, 45°53'45.28"W), xi.1972, coll. N. Papavero & F. Val - 1 male (MZUSP); Guarujá, (23°59'40.56"S, 46°15'24.72"W), i.1942, coll. M. Carrera - 2 females, 1 male (MZUSP); Juquiá, (24°19'17.16"S, 47°38'14.43"W), i.1932, coll. J. Lane - 1 male (MZUSP); Ubatuba, Parque Estadual da Serra do Mar, (23°21'43"S, 44°49'22"W), 21.i.2002, coll. N.W. Perioto & eq. - 1 female (MZUSP); same locality, 24.i.2002, coll. N.W. Perioto & eq. - 1 female, 1 male (MZUSP); Sergipe, Santa Luzia do Itanhy Crasto, (11°22'32.8"S, 37°25'0"W), 1–4.viii.2001, coll. M. T. Tavares & eq. - 1 male (MZUSP).

**Figure 9.**
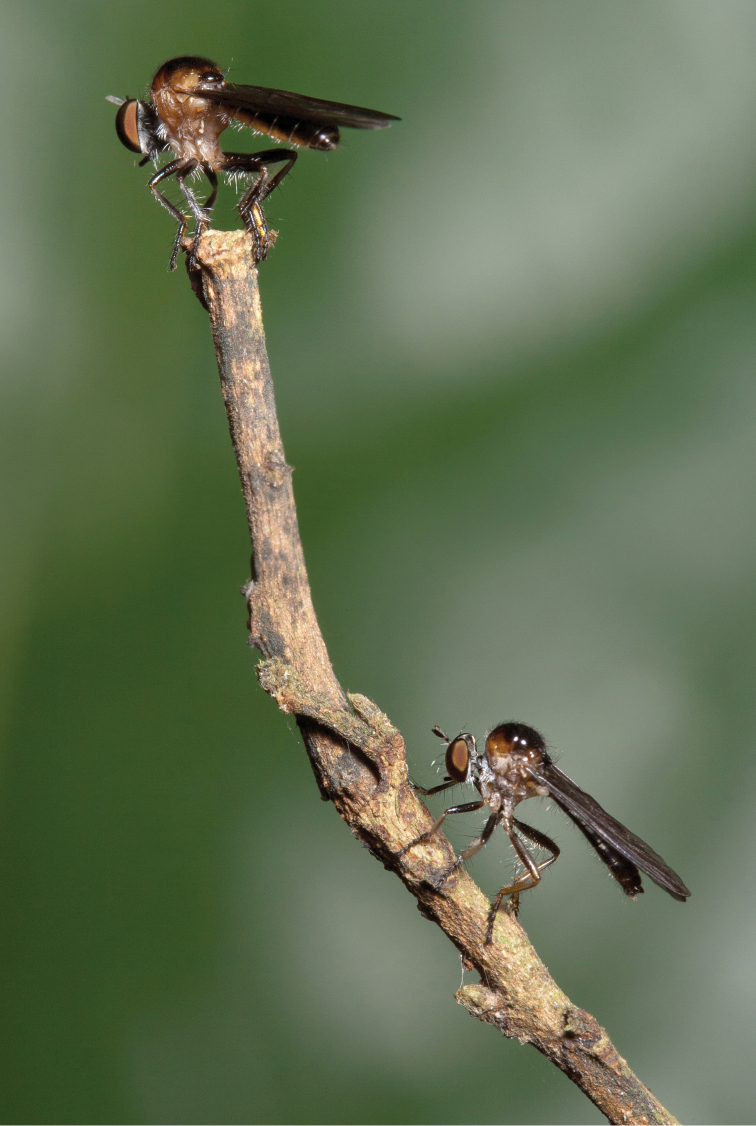
*Oidardis fontenellei* sp. n., Parque Estadual do Rio Doce, Minas Gerais. Female (above) and male (below). (Photo: Guilherme Ide).

**Figure 10.**
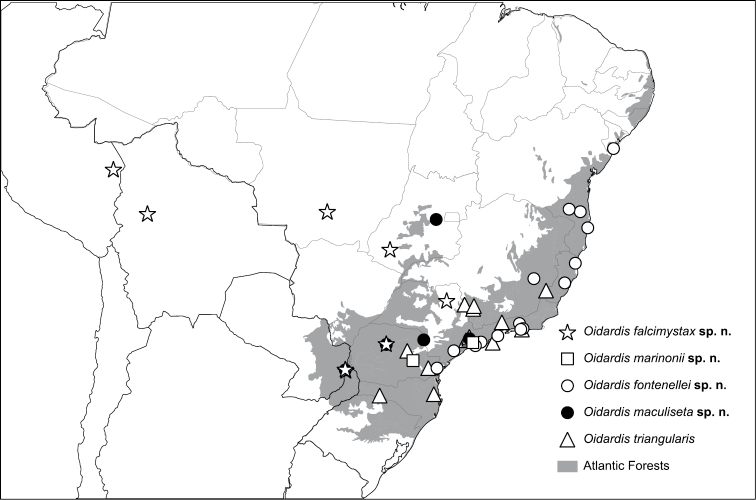
Distribution of *Oidardis* species throughout the Atlantic Rainforest.

#### Oidardis maculiseta sp. n.

http://zoobank.org/03360A78-9C1B-43AB-82BE-C240A012369E

http://species-id.net/wiki/Oidardis_maculiseta

Figures 1E–F , 2C , 3C , 4C , 5C ,6G–I ,7C ,8A ,10

##### Diagnosis.

Leg color pattern: coxae yellow, femora dark-brown dorsally and tibiae yellow dorsally; facial pollinosity golden. Male with dark-brown modified tibial seta, as long as femur, golf-club-shaped with apical 1/4 as a large white lamella with black spot at apex; mid prong of the phallus much longer than lateral prongs; mystax short.

##### Description.

**Holotype.** Male. Body shiny black. Total length, excluding antennae, 5.4 mm; length of thorax, 1.3 mm; length of wing, 4.7 mm; greatest width of abdomen, 1 mm.

**Head, laterally.** Face, between antennal insertion and gibbosity, plane with eye margin; gibbosity slightly prominent, equals ventral 0.26 of face height; dorsal occipital setae dark-brown, lateral occipital setae white, ventral occipital setae white; proboscis 0.48 × the height of head, with numerous white macrosetae ventrally; palpus dark-brown, with yellow setae apically. **Antenna.** Antenna 0.85 × as long as the height of eye, entirely dark-brown, with dark-brown setae and macrosetae; antennal insertion at dorsal 0.2 of head height; scape slightly longer than pedicel, with medium-sized ventral seta, numerous short setae on a row around the segment; pedicel round; postpedicel elongate, 2.1 × length of basal two segments, brown-pollinose, except for silvery-yellow pollinosity on elliptical sensorial area on inner face, with dorsal spine subapical (3/4 length of postpedicel or beyond). **Head, anteriorly.** Head 1.35 × as wide as high; face 0.17 × as wide as head, golden-pollinose; mystax short, comprised of 10 golden macrosetae, and few shorter setae between the rows; facial setae, other than mystax, pale-yellow; frons coppery-pollinose; orbital setae dark-brown; vertex coppery-pollinose; ocellar tubercle coppery-pollinose, as high as vertex, 0.39 × as wide as frons, anterior ocellus 0.14 × as wide as frons by the ocellus position.

**Thorax.** Postpronotal lobe black with yellow spot dorsal to mesothoracic spiracle; scutum shiny black, not punctate, vestiture dark-brown, unequal-sized, reclinate anteriorly and proclinate posteriorly; one; scutellum black, scutellar margin strongly impressed, longest ones slightly longer than scutellum; postalar callosity dark-brown, partly with bright-blue reflections; pleuron shiny dark-brown, with silvery-white pollinosity; setulae on proepisternum, katepisternum, and anepisternum; two anepisternal macrosetae, light-brown; tuft of katatergal macrosetae light-brown; anatergite with golden, hair-like setae.

**Legs.** Coxae orange-yellow; trochanter yellow, with fine yellow setulae; femora yellow, slightly darkened dorsally, covered with short stout yellow setulae dorsally, with dark setae on apical 1/3 dorsally, ventrally with weak yellow setae in two rows, hind femur with 3 long dark-brown ventral macrosetae; anterior four tibiae yellow dorsally and brown ventrally, with white setulae, long yellow macrosetae and long dark-brown macrosetae; hind tibiae yellow, entirely covered by golden setulae, and medium-sized fine dark-brown setae ventrally; modified tibial setae attached to hind tibia at basal 1/3, dark-brown, as long as femur, golf-club-shaped with apical 1/4 as a large white lamella with black spot at apex; tarsi yellow, with stout yellow setae dorsally and densely covered with thick yellow setae, 5th tarsomere with 3 setae apically, opposite the claws and subequal to them; claws yellow on base and black apically; pulvilli yellow and fringed; empodium shorter than claws.

**Wing.** Brownish, darker along upper margin; cell r_1_ with long slightly-concave stalk (2.5 × the length of r-m); crossvein r-m medially in cell d, distal to the end of Sc; cell m_3_ narrowing distally (M_2_ and M_3_ converging by the end of cell m_3_), with stalk slightly longer than r-m, apex of m_3_ and apex of cell d parallel and aligned; crossvein bm-cu short, base of M_3_ and CuA_1_ arranged almost as an “X”; cell cu*p* with stalk shorter than r-m; posterior margin of wing slightly concave at distal half; calypters white, with light-brown margin and fringe of short yellow setae; halter with yellow stem, brown knob.

**Abdomen.** Black, not punctate, with sides diverging posteriorly, T2 1.6 × wider than long; vestiture longer and lighter laterally and ventrally, several light-yellow macrosetae present on lateral margin of T1 and T2. **Male terminalia.** Hypopygium very conspicuous; hypandrium regular-sized (2/3 the width of hypopygium or more), much wider than long, anterior margin straight to slightly convex, posterior margin smoothly convex; gonocoxites partially fused to hypandrium, gonocoxal prolongation thin, smoothly curved inwards, with 2 spines at apex; gonostylus reduced, round, laterally flattened, free, attached to the base of gonocoxite; apex of phallus with three unequal-sized prongs, mid prong much longer than the others; epandrium straight in lateral view; lobes of hypoproct protruding.

**Female.** Total length, excluding antennae, 5.7–7.2 mm, (n=3); length of thorax, 1.5–1.7 mm, (n=3); length of wing, 5.2–6.2 mm, (n=3); greatest width of abdomen, 1.3–1.6 mm, (n=3). Differs from male as follows: gibbosity that equals ventral 0.28–0.3 of face height; proboscis 0.39–0.56 × the height of head; antenna 0.78–0.95 × as long as the height of eye; antennal insertion at dorsal 0.19–0.2 of head height; postpedicel 1.8–2 × length of basal two segments; head 1.4–1.5 × as wide as high; face 0.16 × as wide as head; mystax long (extending beyond the apex of proboscis); ocellar tubercle 0.33 × as wide as frons; anterior ocellus 0.11–0.17 × as wide as frons by the ocellus position; postpronotal lobe dark-brown; proepisternum, anepisternum and katepisternum with golden setulae; trochanter orange-yellow; femora yellow and dark-brown dorsally; femora covered with short, stout brown setulae dorsally; hind femur with 3 long yellow ventral macrosetae; tibiae yellow dorsally and brown ventrally, hind tibia reddish-brown, yellow dorsally on basal 1/3; tibiae with white setulae, long yellow macrosetae, long dark-brown macrosetae, and thick spines; hind tibiae entirely covered by dark-brown setulae; hind tibia with light-brown setulae ventrally, yellow macroseta inserted ventrally on the middle, fine, medium-sized dark-brown setae dorsally, and long, dark-brown macrosetae anterodorsally; modified tibial setae absent; tarsi dark-brown, with claw-like dark-brown setae ventrally, stout dark-brown setae dorsally, and densely covered with thick spine-like dark-brown setae; claws reddish on base and black apically, mid tarsi with yellow-and-black claws; apex of cell m_3_ and apex of cell d angled and unaligned, apex of m_3_ beyond apex of d; posterior margin of wing slightly convex at distal half; halter with orange stem; T2 1.47–1.84 × wider than long; white macrosetae on T1–2; one lateral marginal macrosetae present on T4, T5, T6, and T7. **Female genitalia.** Three spermathecae; reservoirs cylindrical, coiled; spermathecal ducts opening independently at the bursa; genital fork rectangular, U-shaped, arms anteriorly thick, posteriorly slender, divergent; accessory glands oval.

**Morphological variation.** Total length, excluding antennae, 6.0–6.6 mm, (n=4); length of thorax, 1.4–1.5 mm, (n=4); length of wing, 4.9–5.4 mm, (n=4); greatest width of abdomen, 1.0–1.1 mm, (n=4). Some specimens differed from the holotype, as follows: gibbosity that equals ventral 0.23–0.26 of face height; proboscis 0.4–0.57 × the height of head; antenna 0.82–0.86 × as long as the height of eye; antennal insertion at dorsal 0.18–0.24 of head height; postpedicel 1.8–1.9 × length of basal two segments; head 1.37–1.4 × as wide as high; face 0.15–0.17 × as wide as head; mystax comprised of 8–10 macrosetae; ocellar tubercle 0.34–0.37 × as wide as frons; anterior ocellus 0.13–0.16 × as wide as frons by the ocellus position; T2 1.42–1.56 × wider than long.

##### Distribution.

Brazil (Goiás, São Paulo and Paraná).

##### Remarks.

There is a group of females from Fênix (PR) that differs from the paratypes assigned by presenting an oblong postpedicel, dark-brown coxae and homogeneously directed vestiture on the scutum. Therefore, these specimens are not included as paratypes for the species, since they vary in such consistent characters; instead, these specimens are listed under “additional material examined”.

*Oidardis maculiseta* occur mainly in Atlantic Semi-deciduous Forest. As noted above for *Oidardis falcimystax*, they were also found in the Cerrado area, at Corumbá de Goiás, a locality which has a Lower Diptera fauna similar to the Semi-deciduous Forest ecorregion (D. S. Amorim, unpublished data).

##### Etymology.

from the Latin, *macula* = spot, and *seta* = bristle. Refers to the singular morphology of the modified tibial seta.

##### Type-material examined.

**Holotype: Brazil:** Paraná, Fênix, Reserva Estadual ITCF, (23°55'0.05"S, 51°57'38.26"W), 3.xi.1986, coll. Lev. Ent. PROFAUPAR - male (DZUP). **Paratypes: Brazil:** Goiás, Corumbá [de Goiás], Fazenda Monjolinho, (15°55'0.12"S, 48°46'0.12"W), xi.1945, coll. Barretto - 1 male (MZUSP); Paraná, Fênix, Reserva Estadual ITCF, (23°55'0.05"S, 51°57'38.26"W), 6.x.1986, coll. Lev. Ent. PROFAUPAR - 1 male (DZUP); same locality, 20.x.1986, coll. Lev. Ent. PROFAUPAR - 1 male (DZUP); same locality, 3.xi.1986, coll. Lev. Ent. PROFAUPAR - 2 males (DZUP, MZUSP); same locality, 10.xi.1986, coll. Lev. Ent. PROFAUPAR - 3 males (DZUP); same locality, 17.xi.1986, coll. Lev. Ent. PROFAUPAR - 2 males (DZUP, MZUSP); same locality, 24.xi.1986, coll. Lev. Ent. PROFAUPAR - 1 female (DZUP, MZUSP); same locality, 8.xii.1986, coll. Lev. Ent. PROFAUPAR - 2 males (DZUP); same locality, 22.xii.1986, coll. Lev. Ent. PROFAUPAR - 1 female (DZUP); same locality, 29.xii.1986, coll. Lev. Ent. PROFAUPAR - 1 female (DZUP); São Paulo, Barão de Antonina, (23°37'38.07"S, 49°33'40.68"W), i.1946, coll. Barretto - 2 females, 1 male (MZUSP); São Paulo, (23°32'56.19"S, 46°38'19.74"W), xii.1940, coll. M. Carrera (Horto Florestal) - 1 male (MZUSP). **Additional material examined. Brazil:** Paraná, Fênix, Reserva Estadual ITCF, (23°55'0.05"S, 51°57'38.26"W), 20.x.1986, coll. Lev. Ent. PROFAUPAR - 1 female (DZUP); same locality, 27.x.1986, coll. Lev. Ent. PROFAUPAR - 1 females (MZUSP); same locality, 10.xi.1986, coll. Lev. Ent. PROFAUPAR - 1 female (DZUP); same locality, 24.xi.1986, coll. Lev. Ent. PROFAUPAR - 1 female (DZUP); same locality, 8.xii.1986, coll. Lev. Ent. PROFAUPAR - 1 female (DZUP); same locality, 15.xii.1986, coll. Lev. Ent. PROFAUPAR - 1 female (DZUP); Foz do Iguaçu, (25°32'48.83"S, 54°35'17.42"W), 7.xii.1966, coll. Exc. Dep. ZOO - 1 female (DZUP).

#### Oidardis marinonii sp. n.

http://zoobank.org/3AAB47D4-535F-40DB-8496-299F27C96E2A

http://species-id.net/wiki/Oidardis_marinonii

Figures 1G–H , 2D , 3D , 4D , 5D , 6J–L , 7D , 8D , 10

##### Diagnosis.

Pleura yellow; scutum yellow laterally and anteriorly, tergites yellow on lateral margins; scape and pedicel yellow or light brown. Males without modified tibial seta.

##### Description.

**Holotype.** Male. Body yellow and black. Total length, excluding antennae, 7.3 mm; length of thorax, 1.7 mm; length of wing, 6.2 mm; greatest width of abdomen, 1.2 mm.

**Head, laterally.** Face, between antennal insertion and gibbosity, plane with eye margin; gibbosity slightly prominent, equals ventral 0.33 of face height; dorsal occipital setae light-brown, lateral occipital setae light-brown, ventral occipital setae yellow; proboscis 0.52 × the height of head, with a pair of yellow macrosetae ventrally; palpus dark-brown, with yellow setae apically. **Antenna.** Antenna 0.74 × as long as the height of eye, yellow scape and pedicel, dark-brown postpedicel, with dark-brown and yellow setae and macrosetae; antennal insertion at dorsal 0.28 of head height; scape slightly longer than pedicel, with long ventral seta, numerous short setae on a row around the segment; pedicel oval; postpedicel lanceolate, 1.8 × length of basal two segments, golden-pollinose, except for silvery-yellow pollinosity on elliptical sensorial area on inner face, with dorsal spine subapical (3/4 length of postpedicel or beyond). **Head, anteriorly.** Head 1.4 × as wide as high; face 0.17 × as wide as head, silvery-pollinose, on gibbosity only; mystax long (extending beyond the apex of proboscis), comprised of 10 pale-yellow macrosetae, and few shorter setae between the rows; facial setae, other than mystax, pale-yellow; frons silvery-pollinose; orbital setae dark-brown; vertex coppery-pollinose; ocellar tubercle coppery-pollinose, lower than vertex, 0.3 × as wide as frons, anterior ocellus 0.09 × as wide as frons by the ocellus position.

**Thorax.** Postpronotal lobe yellow; scutum shiny dark-brown posteriorly and yellow to light-brown anteriorly, not punctate, vestiture golden, unequal-sized, reclinate anteriorly and proclinate posteriorly; one golden notopleural; scutellum dark-brown, scutellar margin strongly impressed, marginal scutellar macrosetae dark-brown, unequal-sized, longest ones slightly longer than scutellum; postalar callosity light-brown, partly with bright-blue reflections; pleuron yellow, with silvery-white pollinosity; setulae on proepisternum, katepisternum, and anepisternum yellow; two anepisternal macrosetae, plus fine setulae, yellow; tuft of katatergal macrosetae light-brown; anatergite with yellow, hair-like setae.

**Legs.** Coxae yellow; trochanter yellow, with fine yellow setulae; femora reddish-yellow, slightly darkened dorsally, covered with short stout brown setulae dorsally, with dark setae on apical 1/3 dorsally, ventrally with weak yellow setae in two rows and fine yellow setulae apically, hind femur with 3 long yellow ventral macrosetae; anterior four tibiae entirely yellow, with yellow setulae, long yellow macrosetae, long dark-brown macrosetae and thick spines; hind tibia orange, with stout golden setulae apically, white setulae ventrally, long dark-brown macroseta inserted ventrally on the middle, long dark-brown macrosetae posteriorly, and long dark-brown macrosetae anterodorsally; modified tibial setae absent; tarsi reddish-brown, with claw-like dark-brown setae ventrally, stout dark-brown setae dorsally, and densely covered with thick spine-like dark-brown setae, 5th tarsomere with 3 setae apically, opposite the claws and longer than them; claws yellow on base and black apically; pulvilli yellow and fringed; empodium shorter than claws.

**Wing.** Brownish, darker along upper margin; cell r_1_ with short slightly-concave stalk (2 × the length of r-m); crossvein r-m at distal half of cell d, distal to the end of Sc; cell m_3_ narrowing distally (M_2_ and M_3_ converging by the end of cell m_3_), with stalk slightly longer than r-m, apex of m_3_ and apex of cell d parallel, unaligned, apex of m_3_ before apex of d, and unaligned, apex of m_3_ beyond apex of d (right wing) and apex of m_3_ before apex of d (left wing); crossvein bm-cu long, base of M_3_ and CuA_1_ distant from each other and not appearing as an “X”; cell cu*p* with stalk shorter than r-m; posterior margin of wing slightly convex at distal half; calypters pale-yellow, with light-brown margin and fringe of short brown setae; halter with yellow stem, brown knob.

**Abdomen.** Black with yellow lateral margins, punctate, with sides nearly parallel, T2 1.46 × wider than long; vestiture longer and lighter laterally and ventrally, several light-yellow macrosetae present on lateral margin of T1, T2, T3, and T4. **Male terminalia.** Hypopygium very conspicuous; hypandrium regular-sized (2/3 the width of hypopygium or more), much wider than long, anterior margin concave, posterior margin slightly pointed (hypandrium triangular-like); gonocoxites free, gonocoxal prolongation blunt, smoothly curved inwards, with 3 spines at apex; gonostylus reduced, much wider than long, laterally flattened, fused basally to gonocoxite, attached to the base of gonocoxite; apex of phallus with three equal-sized prongs; epandrium straight in lateral view; lobes of hypoproct short.

**Female.** Total length, excluding antennae, 6.2 mm, (n=1); length of thorax, 1.6 mm, (n=1); length of wing, 7.0 mm, (n=1); greatest width of abdomen, 1.1 mm, (n=1). Differs from male as follows: gibbosity that equals ventral 0.3 of face height; proboscis 0.6 × the height of head; antenna 0.71 × as long as the height of eye; antennal insertion at dorsal 0.24 of head height; postpedicel 1.7 × length of basal two segments; head 1.52 × as wide as high; face 0.18 × as wide as head; mystax comprised of 8 macrosetae; tuft of katatergal macrosetae yellow; femora yellow and slightly darkened dorsally, except entirely reddish-brown hind femur; femora ventrally with weak yellow setae in two rows; tibiae with yellow setulae, long yellow macrosetae, and long dark-brown macrosetae; hind tibiae entirely covered by dark-brown setulae, with long, dark-brown macroseta inserted ventrally on the middle and long, dark-brown macrosetae anterodorsally; cell m_3_ parallel-sided distally (M_2_ and M_3_ parallel by the end of cell m_3_), and with stalk as long as r-m; apex of cell m_3_ and apex of cell d angled and unaligned, apex of m_3_ before apex of d; calypters white; halter with milk-coffee knob; abdominal segments narrow, T2 1.88 × wider than long; several macrosetae present on lateral margin of T1, T2, and T3; one lateral marginal macrosetae present on T4. **Female genitalia.** Three spermathecae; reservoirs cylindrical, coiled; spermathecal ducts opening independently at the bursa; genital fork rectangular, U-shaped, arms anteriorly thick, posteriorly slender, divergent; accessory glands distinguishable only for the duct and opening to bursa.

**Morphological variation.** Total length, excluding antennae, 6.2–7.3 mm, (n=5); length of thorax, 1.4–1.8 mm, (n=5); length of wing, 5.7–6.6 mm, (n=5); greatest width of abdomen, 1.0–1.1 mm, (n=4). Some specimens differed from the holotype, as follows: gibbosity that equals ventral 0.26–0.35 of face height; proboscis 0.56–0.64 × the height of head; antenna 0.71–0.82 × as long as the height of eye; light-brown scape and pedicel, dark-brown postpedicel; antennal insertion at dorsal 0.2–0.31 of head height; postpedicel 1.6–2.7 × length of basal two segments; head 1.42–1.53 × as wide as high; face 0.14–0.18 × as wide as head; face golden-pollinose, as a whole; mystax comprised of 8–10 golden macrosetae; ocellar tubercle 0.3–0.34 × as wide as frons; anterior ocellus 0.11 × as wide as frons by the ocellus position; one or two anepisternal macrosetae; apex of cell m_3_ and apex of cell d unaligned, apex of m_3_ before apex of d, or unaligned, apex of m_3_ beyond apex of d; crossvein bm-cu short, base of M_3_ and CuA_1_ arranged almost as an “X”; T2 1.11–1.41 × wider than long.

##### Distribution.

Brazil (São Paulo and Paraná).

##### Remarks.

This species is similar to *Oidardis fontenellei* in general morphology and color pattern. However, since the examined specimens of *Oidardis marinonii* come from a small collection series and are poorly preserved, the observed differences in color of antenna and legs should be used cautiously. The direction of vestiture on the posterior portion of scutum and the absence of the modified tibial seta on males in *Oidardis marinonii* thus become all the more important for separating these species. Nevertheless, the general color pattern—paler pleura and dark scutum—is quite noticeable, even considering preservation issues of the material, and is still reliable when distinguishing both species from others in *Oidardis*.

It is also important to remark that the holotype presents an asymmetry concerning the relative position of cells *d* and *m*_3_, when left and right wings are compared.

*Oidardis marinonii* probably occur in the understory of dense forests, since it is recorded for Cubatão. This locality is situated in the coastal forests of Serra do Mar, a typical Ombrophilous Dense Forest. Therefore, in Ponta Grossa—situated in Araucaria Moist Forest area, but largely covered by grasslands (“campos limpos”)—*Oidardis marinonii* possibly occupies patches of Araucaria woodlands and riparian forests (Marinoni and Dutra 1991, Olson et al. 2001).

##### Etymology.

Honors late Dr. Renato Marinoni, for his efforts on promoting, besides other projects, an important zoological survey in Paraná State (PROFAUPAR), that made available specimens for this species, and many other, to be recognized and described.

##### Type-material examined.

**Holotype: Brazil:** Paraná, Ponta Grossa, Parque Estadual de Vila Velha - IAP, (25°2'37.29"S, 50°14'52.83"W), 22.xii.1986, coll. Lev. Ent. PROFAUPAR - male (DZUP). **Paratypes: Brazil:** Same locality as holotype, 15.xii.1986, coll. Lev. Ent. PROFAUPAR - 1 female, 3 males (DZUP, MZUSP); same locality, 22.xii.1986, coll. Lev. Ent. PROFAUPAR - 5 males (DZUP, MZUSP); same locality, 29.xii.1986, coll. Lev. Ent. PROFAUPAR - 3 males (DZUP); São Paulo, Cubatão, (23°53'44.02"S, 46°25'32.28"W), 15.xii.1955, coll. Pereira, Martinez, Werner & d’Andretta - 1 male (MZUSP).

## Supplementary Material

XML Treatment for Oidardis falcimystax

XML Treatment for Oidardis fontenellei

XML Treatment for Oidardis maculiseta

XML Treatment for Oidardis marinonii
